# Influence of Poor Oral Health on Physical Frailty: A Population‐Based Cohort Study of Older British Men

**DOI:** 10.1111/jgs.15175

**Published:** 2017-12-20

**Authors:** Sheena E. Ramsay, Efstathios Papachristou, Richard G. Watt, Georgios Tsakos, Lucy T. Lennon, A. Olia Papacosta, Paula Moynihan, Avan A. Sayer, Peter H. Whincup, S. Goya Wannamethee

**Affiliations:** ^1^ Institute of Health and Society Newcastle University Newcastle upon Tyne United Kingdom; ^2^ Department of Primary Care and Population Health Institute of Epidemiology and Health Care University College of London London United Kingdom; ^3^ Institute of Education University College of London London United Kingdom; ^4^ Department of Epidemiology and Public Health Institute of Epidemiology and Health Care University College of London London United Kingdom; ^5^ Centre for Oral Health Research and School of Dental Sciences Newcastle University Newcastle upon Tyne United Kingdom; ^6^ Institute of Neuroscience Newcastle University Newcastle upon Tyne United Kingdom; ^7^ Biomedical Research Centre National Institute for Health Research Newcastle University and Newcastle upon Tyne Hospitals NHS Foundation Trust Newcastle upon Tyne United Kingdom; ^8^ Population Health Research Institute St George's University of London London United Kingdom

**Keywords:** frailty, oral health, longitudinal investigations

## Abstract

**Objectives:**

To investigate the associations between objective and subjective measures of oral health and incident physical frailty.

**Design:**

Cross‐sectional and longitudinal study with 3 years of follow‐up using data from the British Regional Heart Study.

**Setting:**

General practices in 24 British towns.

**Participants:**

Community‐dwelling men aged 71 to 92 (N = 1,622).

**Measurements:**

Objective assessments of oral health included tooth count and periodontal disease. Self‐reported oral health measures included overall self‐rated oral health; dry mouth symptoms; sensitivity to hot, cold, and sweet; and perceived difficulty eating. Frailty was defined using the Fried phenotype as having 3 or more of weight loss, grip strength, exhaustion, slow walking speed, and low physical activity. Incident frailty was assessed after 3 years of follow‐up in 2014.

**Results:**

Three hundred three (19%) men were frail at baseline (aged 71–92). Having fewer than 21 teeth, complete tooth loss, fair to poor self‐rated oral health, difficulty eating, dry mouth, and more oral health problems were associated with greater likelihood of being frail. Of 1,284 men followed for 3 years, 107 (10%) became frail. The risk of incident frailty was higher in participants who were edentulous (odds ratio (OR) = 1.90, 95% confidence interval (CI) = 1.03–3.52); had 3 or more dry mouth symptoms (OR = 2.03, 95% CI = 1.18–3.48); and had 1 (OR = 2.34, 95% CI = 1.18–4.64), 2 (OR = 2.30, 95% CI = 1.09–4.84), or 3 or more (OR = 2.72, 95% CI = 1.11–6.64) oral health problems after adjustment for age, smoking, social class, history of cardiovascular disease or diabetes mellitus, and medications related to dry mouth.

**Conclusion:**

The presence of oral health problems was associated with greater risks of being frail and developing frailty in older age. The identification and management of poor oral health in older people could be important in preventing frailty.

## Abstract 2

See related editorial by https://doi.org/10.1111/jgs.15253.

The United Kingdom, in common with many other developed countries, is undergoing rapid demographic changes, with dramatic increases in the number of older adults. The numbers of people aged 65 and older and 85 and older in England and Wales are projected to increase by 25% and 50%, respectively, by 2033.^1^ The health and well‐being of older adults is therefore a public health priority. Oral health problems are widely prevalent health conditions in older adults, and with population aging, the global burden of oral health problems has increased over the last 20 years.^2^ Oral health problems in older people include excessive tooth loss, periodontal (gum) disease, dental caries (decay), and perceived dry mouth. In the United Kingdom, more than 60% of older adults have periodontal disease, only approximately 40% have functional dentition (defined as ≥21 teeth), and more than one‐third have dry mouth.^3^, ^4^ These oral health problems have significant effects on eating and swallowing, nutritional intake, speaking, and smiling and thus affect several aspects of health and well‐being.^5^ Tooth loss and periodontal disease are also found to be associated with greater risks of morbidity, physical and cognitive decline, and mortality.^6^, ^7^, ^8^

Studies suggest that poor oral health is also associated with greater risk of frailty, which is a major healthcare challenge in aging populations.^9^, ^10^ Frailty is a state of vulnerability in older age to adverse outcomes including functional decline, hospitalization, disability, long‐term care, and death.^10^, ^11^ Although an association has been found between poor oral health (e.g., tooth loss) and frailty, studies so far mostly have few oral health measures (typically, presence or absence of teeth) in relation to individual aspects of frailty, and have mostly been cross‐sectional.^9^ Three previous studies with a composite measure of frailty investigating associations with oral health were cross‐sectional.^12^, ^13^, ^14^ One recent study of 237 older Mexican adults investigated the prospective association between oral health and frailty and found tooth loss and periodontal disease to be associated with greater risk of incident frailty.^15^ Further studies in different and larger study populations are needed to corroborate and establish the influence of oral health longitudinally on the development of frailty. Such investigations are needed to identify the role of oral health in reducing the burden of frailty in older age. In a unique national study comprising a sample of community‐dwelling British men aged 71 to 92, we prospectively investigated whether objective and subjective oral health measures are associated with frailty over 3 years of follow‐up. We also examined whether these associations were independent of smoking, socioeconomic position, and history of cardiovascular disease and diabetes.

## Methods

The British Regional Heart Study (BRHS) is a prospective cohort comprising a socially and geographically representative sample of 7,735 British men recruited from general practices in 24 towns across Britain initially examined in 1978 to 1980 at age 40 to 59.^16^ From 2010 to 2012, all surviving men then aged 71 to 92 were invited to attend a reexamination. All relevant local research ethics committees provided ethical approval. All men provided written informed consent to participate in the investigations, which were conducted in accordance with the Declaration of Helsinki. Participants (N = 1,722) underwent a physical examination including anthropometric measurements (height, weight, waist circumference) and physical performance assessments including a walking test (time taken, in seconds, to walk 3 m at normal walking pace), and grip strength hydraulic hand dynamometer (Jamar Hydraulic Hand Dynamometer Model J00105).^16^ Grip strength was measured 3 times for each hand, and the best of 6 readings was used for the analysis. Participants also completed a questionnaire at the time of examination or by mail if they did not attend (n = 2,147) that included information such as their medical history and lifestyle factors. In 2014, a postal questionnaire with medical, social, and health‐related questions was sent to participants as part of the on‐going follow‐up of the study. The investigations in the present study are based on the assessments made at age 71 to 92 in 2010–12, with follow‐up until 2014.

### Oral Health Markers

The physical examination at age 71 to 92 in 2010–12 included an oral health assessment comprising a count of natural teeth and two measures of periodontal conditions on 6 index teeth (three in the upper and three in the lower jaw)—periodontal pocket depth (the gap between gums and tooth) and loss of attachment (the distance between the point at which the gum is attached and the “neck” of the tooth where the gum is attached in a healthy tooth).^17^ Questionnaires included questions on self‐reported oral health measures, including overall self‐rated oral health (excellent, good, or fair to poor);^18^ dry mouth (based on the validated Xerostomia Inventory Scale);^19^ sensitivity to hot, cold, and sweet; and difficulty eating food.

### Frailty

Frailty status at age 71 to 92 in 2010–12 was based on the Fried frailty phenotype using data from both questionnaires and the physical assessment.^11^, ^20^ This included unintentional weight loss (assessed as ≥5% decrease in self‐reported weight that was reported to be unintentional); exhaustion (response of “no” to question “Do you feel full of energy?”); weakness (assessed as lowest fifth of grip strength distribution); and slow walking speed (lowest fifth of walking speed) according to the frailty phenotype criteria.^11^, ^20^ If walking speed was unavailable, we used information on self‐reported slow walking pace (being unable to walk more than few steps or <200 yards or difficulty walking across a room) or low physical activity (self‐report of being less or much less active than an average man). Presence of 3 or more of these components was defined as frailty.

Frailty status at the 3‐year follow‐up in 2014 was based on information from postal questionnaires (response rate 64%). Frailty phenotype was based on subjective measures of the frailty components. This measure of frailty has been found to be as predictive of established adverse outcomes (disability, falls, death) as the frailty measure using objective measures in our study.^21^ Exhaustion and low physical activity were assessed in the same way as at baseline (aged 71–92), with questions on exhaustion (Do you feel full of energy?) and physical activity (less or much less active than an average man). Grip strength was based on participants' rating of their grip strength compared with that of other people their age; response of fair or poor was classified as low grip strength. Slow walking speed was based on self‐report of usual walking pace. Participants who rated their walking pace as slow were classified as having slow walking speed. Weight loss was based on self‐report of a decrease in weight in the last 4 years.

### Covariates

Socioeconomic status was based on the longest‐held occupation recorded at study entry (aged 40–59) and included six social class groups (I, II, III nonmanual, III manual, IV, V).^22^ For the purposes of this study, social classes I, II, and III nonmanual were grouped as nonmanual, and III manual, IV, and V were grouped as manual social class. Detailed questions on smoking and medical history were included in the questionnaire in 2010–12. Smoking status was categorized as current smoker, long‐term exsmoker (gave up smoking before 1983), recent exsmoker, and never smoker. History of coronary heart disease (CHD) was based on report of a doctor's diagnosis of angina pectoris, heart attack (coronary thrombosis, myocardial infarction), or heart failure. History of diabetes was based on a doctor's diagnosis of diabetes or a fasting glucose level greater than 7 mmol/L. Regular use of prescribed medications with xerostomia (dry mouth) as a side effect was identified from questionnaires and included antimuscarinics (anticholinergics), antidepressants (tricyclics, selective serotonin reuptake inhibitors), alpha‐blockers, antihistamines, antipsychotics, baclofen, bupropion, clonidine, 5HT1 agonists, opioids, tizanidine, and diuretics.^23^ Participants were categorized in 1 of 3 groups as taking 0, 1, or 2 or more of these medications. The use of medications with xerostomia as a side effect was closely related to report of dry mouth symptoms (67% of those taking ≥1 of these reported having at least one dry mouth symptom, *P* = .001).

### Statistical Analysis

Associations between oral health measures and frailty were examined cross‐sectionally (at age 71–92 in 2010–12) and prospectively (incident frailty at 3‐year follow‐up in 2014) using logistic regression models with nonfrail as the reference group. Two measures of tooth loss were used (3‐category variable of ≥21 (the minimum considered for functional dentition), 1–20, and 0 (edentulism); 0 (edentulism). Two measures of periodontal conditions were examined (>20% sites with periodontal pockets >3.5 mm as a proportion of number of sites examined; >20% sites with loss of attachment >5.5 mm as a proportion of number of sites examined). Self‐rated oral health was grouped into excellent or good versus fair or poor; dry mouth symptoms were categorized as 0, 1 to 2, and 3 or more symptoms; and sensitivity to hot, cold, and sweet and difficulty eating were binary (yes or no). A cumulative measure of poor oral health was created combining complete tooth loss; fair or poor self‐rated oral health; dry mouth; sensitivity to hot, cold, or sweet; and difficulty eating and categorized as 0, 1, 2, or 3 or more oral health problems. Adjusted odds ratios (ORs) with 95% confidence intervals (CIs) were computed after adjustment for age, social class, smoking status, history of diabetes, history of cardiovascular disease (CVD), and use of medications with dry mouth as a side effect. For the adjustments, age was fitted as a continuous variable. Social class (2 levels); smoking status (4 levels), history of CVD or diabetes (2 levels), and use of medications (3 levels) were fitted as categorical variables in the regression models. In the models for incident frailty, prevalent cases of frailty at baseline (2010–12) were excluded (n = 168, 14%). All analyses were performed using Stata/SE version 14 (Stata Corp., College Station, TX).

## Results

In 2010–12, 1,722 participants aged 71 to 92 attended a reexamination (55% response rate), of whom 1,622 had complete data on frailty status; 2147 men completed questionnaires (68% response rate). The prevalence of edentulism was 20%, and 64% had fewer than 21 teeth. In terms of periodontal conditions, 25% had loss of attachment greater than 5.5 mm and 29% periodontal pocket depth greater than 3.5 mm. For self‐reported oral health measures, 11% reported sensitivity to hot, cold, or sweet; 33% had 1 dry mouth symptom, and 29% had 2 or more; 34% rated their oral health as fair or poor; and 11% reported difficulty eating. The prevalence of frailty was 19% (n = 303). After an average of 3 years of follow‐up, 1,284 men completed a postal questionnaire in 2014 (64% response rate). Based on these follow‐up data, there were 107 (10%) cases of incident frailty over the follow‐up period.

Table 1 summarizes the baseline characteristics of participants aged 71 to 92 according to their frailty status. Participants identified as frail were older (on average) (*P* < .001) and more likely to be in the manual social class (*P* = .03), have a history of diabetes or CVD (*P* < .001), and be currently prescribed 2 or more medications with dry mouth as a side effect (*P* < .001) than those identified as nonfrail. Participants who were frail had higher levels of each of the frailty components (weakness, exhaustion, weight loss, low physical activity, slow walking speed) than nonfrail participants (all *P* < .001; results presented in Supplementary Tables S1 and S2).

**Table 1:** Baseline Characteristics According to Frailty Status in a Population‐Based Sample of British Men Aged 71 to 92 (N = 1,622)

Characteristic	Nonfrail, n = 1,319	Frail, n = 303	P‐Value
Age, mean ± standard deviation	77.9 ± 4.3	80.5 ± 5.4	<.001
History of diabetes or cardiovascular disease, n (%)	518 (39)	180 (59)	<.001
Social class, n (%)			
Nonmanual	703 (55)	139 (48)	.03
Manual	581 (45)	152 (52)	
Smoking, n (%)			
Never	521 (40)	94 (31)	.03
Long‐term exsmoker (gave up before 1983)	573 (43)	148 (49)	
Recent exsmoker	178 (14)	52 (17)	
Current smoker	46 (3)	9 (3)	
Number of prescribed medications with xerostomia (dry mouth) as a side effect, n (%)			
0	774 (59)	141 (47)	<.001
1	420 (32)	115 (38)	
≥2	125 (9)	47 (16)	

Table 2 shows the cross‐sectional associations between oral health measures and frailty at baseline (aged 71–92). In age‐adjusted models, men who were edentulous and had fair or poor self‐rated oral health, difficulty eating, and dryness of mouth were more likely to be frail. Associations between frailty and edentulism (OR = 1.63, 95% CI = 1.18–2.23), fair or poor self‐rated oral health (OR = 1.56, 95% CI = 1.18–2.07), and dry mouth symptoms (OR for ≥3 symptoms = 2.50, 95% CI = 1.77–3.53) remained significant after further adjustment for smoking, social class, history of CVD or diabetes, and medication use. Having more oral health problems was also significantly associated with being frail in the fully adjusted model (OR per additional oral health problem = 1.32, 95% CI = 1.15–1.52; *P* for trend <.001).

**Table 2:** Likelihood of Frailty (n = 303) According to Oral Health Measures in a Population‐Based Study of 1,622 British Men Aged 71 to 92 (N = 1,622)

Oral Health Measures	n (%)	Age Adjusted	Fully Adjusted^a^
Number of teeth			
≥21 (n = 563)	75 (13)	1.00	1.00
<21 (n = 1,003)	218 (22)	1.55 (1.15–2.08)^b^	1.32 (0.96–1.81)
Number of teeth			
≥1 (n = 1,251)	200 (16)	1.00	1.00
0 (n = 315)	93 (30)	1.84 (1.37–2.48)^b^	1.63 (1.18–2.23)^b^
Number of teeth			
≥21 (n = 563)	75 (13)	1.00	1.00
1–20 (n = 688)	125 (18)	1.30 (0.95–1.78)	1.15 (0.83–1.61)
0 (n = 315)	93 (30)	2.16 (1.51–3.08)^b^	1.78 (1.21–2.63)^b^
*P* for trend		<.001	.002
Loss of attachment (percentage of sites with >5.5 mm)			
<20% (n = 896)	130 (15)	1.00	1.00
≥20% (n = 291)	47 (16)	1.07 (0.74–1.55)	0.98 (0.67–1.44)
Periodontal pocket depth (percentage of sites with >3.5 mm)			
<20% (n = 841)	119 (14)	1.00	1.00
≥20% (n = 346)	58 (17)	1.25 (0.88–1.78)	1.13 (0.78–1.63)
Self‐rated oral health			
Good or excellent (n = 1,022)	157 (15)	1.00	1.00
Fair or poor (n = 538)	128 (24)	1.66 (1.27–2.16)^b^	1.56 (1.18–2.07)^b^
Difficulty eating			
No (n = 1,443)	254 (18)	1.00	1.00
Yes (n = 179)	49 (27)	1.55 (1.07–2.24)^b^	1.37 (0.93–2.03)
Sensitivity to hot, cold, or sweet			
No (n = 1,158)	204 (18)	1.00	1.00
Yes (n = 359)	68 (19)	1.23 (0.90–1.69)	1.22 (0.88–1.69)
Number of dry mouth symptoms			
0 (n = 591)	71 (12)	1.00	1.00
1–2 (n = 515)	90 (17)	1.59 (1.13–2.24)^b^	1.58 (1.10–2.26)^b^
≥3 (n = 454)	124 (27)	2.59 (1.86–3.60)^b^	2.50 (1.77–3.53)^b^
Per additional dry mouth symptom		1.24 (1.17–1.31) *P* for trend <.001	1.23 (1.16–1.30) *P* for trend <.001
Number of cumulative oral health problems^c^			
0 (n = 347)	45 (13)	1.00	1.00
1 (n = 745)	124 (17)	1.21 (0.83–1.76)	1.06 (0.71–1.56)
2 (n = 373)	82 (22)	1.64 (1.09–2.47)^b^	1.38 (0.91–2.11)
≥3 (n = 157)	52 (33)	2.86 (1.79–4.56)^b^	2.32 (1.42–3.81)^b^
Per additional oral health problem		1.39 (1.22–1.59) *P* for trend<.001	1.32 (1.15–1.52) *P* for trend <.001

a Adjusted for age, social class, smoking status, history of diabetes or cardiovascular disease, and use of medication with dry mouth as a side effect.

b *P* < .05.

c Includes <21 teeth; difficulty eating; symptoms of dry mouth; and sensitivity to hot, cold, or sweet.

In age‐adjusted models, the risk of becoming frail over the 3‐year follow‐up period was greater in participants who were edentulous (OR = 1.78, 95% CI = 1.10–2.88) and those reporting fair or poor oral health (OR = 1.65, 95% CI = 1.08–2.52) (Table 3). Periodontal measures of loss of attachment and periodontal pocket depth were not significantly associated with incident frailty. Problems of sensitivity to hot, cold, or sweet (OR = 1.13, 95% CI = 0.69–1.84) and self‐reported difficulty eating (OR = 1.69, 95% CI = 0.96–2.95) were also not significantly associated with incident frailty. Participants with 1 or 2 (OR = 1.83, 95% CI = 1.09–3.07) or 3 or more dry mouth symptoms (OR = 2.20, 95% CI = 1.30–3.73) were at a higher risk of incident frailty than those without. The risk of incident frailty was slightly greater for every additional dry mouth symptom (OR = 1.18, 95% CI = 1.07–1.29; *P* for trend = .001). Finally, the risk of incident frailty was higher in participants with more oral health problems (1‐item increase in number of oral health problems: OR = 1.36, 95% CI = 1.10–1.67; *P* for trend = .005). After further adjustments for covariates, the risk of developing frailty remained higher in participants who were edentulous (OR = 1.90, 95% CI = 1.03–3.52); had 3 or more dry mouth symptoms (OR = 2.05, 95% CI 1.19–3.51); and had 1 (OR = 2.31, 95% CI = 1.17–4.58), 2 (OR = 2.30, 95% CI = 1.09–4.85), or 3 or more (OR = 2.71, 95% CI = 1.11–6.62) oral health problems.

**Table 3:** Likelihood of Incident Frailty (n = 107) According to Oral Health Measures in a Population‐Based Study of British Men Aged 71 to 92 (N = 1,054) Followed for 3 Years

Oral Health Measure	n (%)	Age Adjusted	Fully Adjusted^a^
		Odds Ratio (95% Confidence Interval)	
Number of teeth			
≥21 (n = 403)	28 (7)	1.00	1.00
<21 (n = 618)	75 (12)	1.64 (1.03–2.60)^b^	1.46 (0.89–2.38)
Number of teeth			
≥1 (n = 852)	75 (9)	1.00	1.00
0 (n = 169)	28 (17)	1.78 (1.10–2.88)^b^	1.59 (0.96–2.66)
Number of teeth			
≥21 (n = 403)	28 (7)	1.00	1.00
1–20 (n = 449)	47 (10)	1.43 (0.87–2.35)	1.31 (0.78–2.21)
0 (n = 169)	28 (17)	2.20 (1.25–3.90)	1.90 (1.03–3.52)
*P* for trend		.002	.02
Loss of attachment (% of sites with >5.5 mm)			
<20% (n = 624)	48 (8)	1.00	1.00
≥20% (n = 194)	20 (10)	1.24 (0.71–2.16)	1.09 (0.61–1.97)
Periodontal pocket depth (percentage of sites with >3.5 mm)			
<20% (n = 586)	48 (8)	1.00	1.00
≥20% (n = 232)	20 (9)	1.04 (0.60–1.80)	1.04 (0.59–1.84)
Self‐rated oral health			
Good or excellent (n = 688)	57 (8)	1.00	1.00
Fair or poor (n = 329)	44 (13)	1.65 (1.08–2.52)^b^	1.55 (0.99–2.41)
Difficulty eating			
No (n = 945)	89 (9)	1.00	1.00
Yes (n = 109)	18 (17)	1.69 (0.96–2.95)	1.38 (0.75–2.53)
Sensitive to hot, cold, or sweet			
No (n = 762)	74 (10)	1.00	1.00
Yes (n = 236)	24 (10)	1.13 (0.69–1.84)	1.07 (0.63–1.80)
Number of dry mouth symptoms			
0 (n = 401)	27 (7)	1.00	1.00
1–2 (n = 346)	39 (11)	1.83 (1.09–3.07)^b^	1.63 (0.96–2.78)
≥3 (n = 274)	38 (14)	2.20 (1.30–3.73)^b^	2.05 (1.19–3.51)^b^
Per additional dry mouth symptom		1.18 (1.07–1.29) *P* for trend=.001	1.17 (1.06–1.28) *P* for trend=.002
Number of cumulative oral health problems^c^			
0 (n = 246)	11 (4)	1.00	1.00
1 (n = 483)	53 (11)	2.48 (1.26–4.86)^b^	2.31 (1.17–4.58)^b^
2 (n = 235)	29 (12)	2.74 (1.33–5.65)^b^	2.30 (1.09–4.85)^b^
≥3 (n = 90)	14 (16)	3.45 (1.49–7.99)^b^	2.71 (1.11–6.62)^b^
Per additional oral health problem		1.36 (1.10–1.67) *P* for trend = .005	1.26 (1.01–1.58) *P* for trend = .04

a Adjusted for age, social class, smoking status, history of diabetes or cardiovascular disease, and use of medication with dry mouth as a side effect.

b *P* < .05.

c Includes <21 teeth; difficulty eating; symptoms of dry mouth; and sensitivity to hot, cold, or sweet.

## Discussion

Our study in a population‐based sample of British men aged 71 to 92 showed that various aspects of poor oral health were associated with being frail and with incident frailty in older age. In our longitudinal investigations in 1,054 older adults, we found that complete tooth loss, dry mouth, and cumulative oral health problems were, in particular, associated with incidence of frailty independent of socioeconomic factors and comorbidities. These findings highlight the importance of oral health in older populations and the potential contribution of poor oral health to developing frailty.

The influence of poor oral health in older age on frailty is not well established. To our knowledge, the only previous prospective study recently published on oral health and frailty was in Mexican older adults.^15^ Similar to our findings, that study found an association between tooth loss and frailty, but unlike the previous study, we did not find periodontal disease markers to be associated with incident frailty, which could be because the measures of periodontal disease in our study were limited to 6 index teeth and possible differences in the study populations.

In our prospective analyses, we found that complete tooth loss, poor self‐rated oral health, and dry mouth were associated with incident frailty over 3 years of follow‐up, although the associations with poor self‐rated oral health were attenuated after adjustment for socioeconomic factors and comorbidities. The influence of dry mouth remained significantly associated with incident frailty even after adjustments for covariates. Dry mouth in older age is often a consequence of polypharmacy, particularly as a side effect of the use of antidepressants, antihypertensive medications (alpha‐ and beta‐blockers, diuretics, calcium channel blockers, angiotensin‐converting enzyme inhibitors).^24^, ^25^ Dry mouth affects oral health–related quality of life and denture‐related problems (sores and ulcers) and has important influences on eating and swallowing functions. It is possible that the influence of tooth loss and dry mouth on frailty is because of their effect on nutritional status.^26^ Although self‐reported difficulty eating itself was not associated with frailty, it is not a robust marker of nutritional status, which needs to be further explored as a possible mediator using robust validated methods. It is also possible that underlying comorbidities mediate the relationship between dry mouth and frailty. Although we adjusted for diabetes, CVD, and medication use, the possibility of residual confounding remains. Another important finding of our study that has not been previously reported is the association between a composite or cumulative measure of oral health problems and frailty; we found that having more oral health problems was associated with greater risk of incident frailty, which remained significant on full adjustment.

### Strengths and Limitations

A particular strength of our study is that we prospectively investigated associations between a range of oral health measures and incidence of frailty in a representative sample of older British men. The oral health measures used in our study included objective assessments of tooth count and periodontal disease and self‐reported measures of self‐rated oral health and dry mouth. Self‐rated oral health is known to be a strong marker of oral disease,^18^, ^27^, ^28^ and dry mouth is prevalent, affecting more than one‐third of older adults.^17^, ^25^ We also created a composite score to capture the combined effect of oral health problems in older adults. Limitations of our study are that our sample comprised older men who were mostly white Europeans. Therefore, our results have limited generalizability to older women and other ethnic groups. The moderate response rate of 55% for the physical examination at baseline is likely to have excluded individuals with worse health. As in previous examinations, nonresponders tend to be older and more likely to be in a manual social class groups and have poor or fair self‐rated oral health. It is therefore possible that the role of socioeconomic position and comorbidities was not fully accounted for in our adjustments for the associations between oral health measures and frailty. Our outcome measure of frailty was based on self‐reported measures of frailty components, as opposed to the baseline measure of frailty, which included objective measures of grip strength and walking speed. It is possible that our outcome measures underestimated the incidence of frailty. Nevertheless, we have shown that this frailty measure is robust and is just as predictive of known adverse outcomes of frailty (disability, falls, death) as frailty measures comprising objective components.^21^

## Conclusions

Our findings highlight the importance of oral health problems that are not only associated with the presence of physical frailty, but may also influence the development of frailty in older age. Our findings particularly highlight the importance of tooth loss, dry mouth, and cumulative oral health problems, all of which were independently associated with incident frailty. Further research is needed to understand mechanisms underlying associations between oral health and frailty, for example to explore whether the association is mediated through nutrition and inflammation. Although causal associations cannot be fully established from our study, our findings suggest that dry mouth or accumulation of oral health problems could be powerful markers and predictors of frailty in older people. Increasing interest in identifying frail older people has led to tools such as the Frailty Index, which includes deficits such as comorbidities, poor physical function, and sensory impairments,^10^ but oral health is underrecognized in the assessment and care of older people. Markers of poor oral health could be useful indicators of frailty and valuable additions to health screening assessments used in older people. Further research is needed to develop simple markers of oral health that could be used widely in assessments of older people. Poor oral health could also be important as a modifiable risk factor for frailty through its effect on oral intake and nutritional status. This needs to be investigated in intervention studies.

## Supporting information


**Table S1.** Cross‐tabulation between prevalent frailty status and individual frailty components in a population‐based study of 1,622 British men aged 71 to 92 in 2010–12
**Table S2.** Cross‐tabulation between prevalent frailty status and individual self‐reported frailty components in a population‐based study of 1,655 British men aged 74–95 years in 2014
