# Supplementary material for: Influence of Poor Oral Health on Physical Frailty: A Population‐Based Cohort Study of Older British Men
Source: J Am Geriatr Soc. 2017 Dec 20;66(3):473–9. doi: 10.1111/jgs.15175 (PMC5887899; doi:10.1111/jgs.15175)
Supplement: Supplementary file 1 — Table S1. Cross‐tabulation between prevalent frailty status and individual frailty components in a population‐based study of 1,622 British men aged 71 to 92 in 2010–12 Table S2. Cross‐tabulation between prevalent frailty status and individual self‐reported frailty components in a population‐based study of 1,655 British men aged 74–95 years in 2014 [file JGS-66-473-s001.docx]

Supplementary for online publication.

Table S1. Cross-tabulation between prevalent frailty status and individual frailty components in a population-based study of 1,622 British men aged 71-92 years in 2010-12

|  | **Non-frail (n=1,319; 81%)** | **Frail (n=303; 19%)** | **p-value** |
| --- | --- | --- | --- |
| Weakness (n=342; 21%) | 200 (15%) | 142 (47%) | <.001 |
| Exhaustion (n=877; 54%) | 592 (45%) | 285 (94%) | <.001 |
| Weight loss (n=183; 11%) | 85 (6%) | 98 (33%) | <.001 |
| Low physical activity (n=521; 32%) | 254 (19%) | 267 (88%) | <.001 |
| Slow walking speed (n=352; 22%) | 120 (9%) | 232 (78%) | <.001 |

Table S2. Cross-tabulation between prevalent frailty status and individual self-reported frailty components in a population-based study of 1,655 British men aged 74-95 years in 2014

|  | **Non-frail (n=1,393; 84%)** | **Frail (n=262; 16%)** | **p-value** |
| --- | --- | --- | --- |
| Weakness (n=322; 20%) | 157 (11%) | 118 (66%) | <.001 |
| Exhaustion (n=200; 12%) | 61 (4%) | 139 (53%) | <.001 |
| Weight loss (n=354; 22%) | 242 (18%) | 112 (44%) | <.001 |
| Low physical activity (n=522; 32%) | 285 (21%) | 237 (90%) | <.001 |
| Slow walking speed (n=521; 33%) | 281 (21%) | 240 (94%) | <.001 |
